# Alcohol-related Consequences: Factor Structure and Associations With Trait Mindfulness and Drinking Motivations

**DOI:** 10.1007/s12529-024-10300-7

**Published:** 2024-06-04

**Authors:** Lauren M. Carney, Crystal L. Park, Beth Russell

**Affiliations:** 1 Dept. of Population Health Science and Policy, Icahn School of Medicine at Mount Sinai, 1 Gustave L. Levy Place, New York, NY 10029 USA; 2 Dept. of Psychological Sciences, University of Connecticut, 406 Babbidge Road, Unit 1020, Storrs, CT 06269 USA; 3 Dept. of Human Development and Family Sciences, University of Connecticut, 348 Mansfield Rd, Storrs, USA

**Keywords:** Trait mindfulness, Motivations to drink, Alcohol-related consequences, Drinking to cope, Collegiate substance use

## Abstract

**Background:**

This study aimed to determine if motivations to use alcohol (coping and social motivations) mediate the relationship between trait mindfulness and a variety of alcohol-related consequences and to determine if the relationship between motivations to use alcohol and alcohol-related consequences is moderated by alcohol use. We determined the factor structure of positive and negative consequences of alcohol use and used this structure as outcomes across eight moderated mediation models.

**Methods:**

Data were obtained from 296 undergraduate students to confirm the alcohol-related consequences factor structure and to test eight moderated-mediation models.

**Results:**

Four alcohol-related consequences scales (romantic/sexual, positive, mild negative, and severe negative consequences) were confirmed. The motive of drinking to cope significantly mediated the relationship between trait mindfulness and all four of the alcohol-related consequences scales. Drinking to socialize did not significantly mediate the relationship between trait mindfulness and all of the alcohol-related consequences scales.

**Conclusions:**

The identified four-factor structure suggests that alcohol-related consequences should be assessed in a more specific manner. Additionally, different motivations for alcohol use relate differentially to trait mindfulness and different alcohol-related consequences; drinking to cope is particularly problematic for this population. Future research on the usefulness of promoting mindfulness to reduce problematic drinking appears warranted.

**Supplementary Information:**

The online version contains supplementary material available at 10.1007/s12529-024-10300-7.

## Introduction

Alcohol consumption is prevalent on college campuses; nearly half of full-time college students report drinking alcohol in the past month and nearly 39% report engaging in binge drinking (i.e., consuming 5 drinks or more on one occasion for males and 4 drinks or more for females) [1]. Binge drinking in college has been identified as a serious public health hazard for college students [1] and is related to a greater risk of experiencing alcohol-related consequences [2] as well as later alcohol use disorder diagnosis [3]. Alcohol-related consequences for college students can range from to injuries, legal consequences, and even death [1], and one study found that half of age 19-20 year-old drinkers and 75% of age 19-20 year-old binge drinkers reported experiencing some type of negative alcohol-related consequence [2].

Research suggests that motivations to drink alcohol, or the factors and antecedents that impact drinking, may predict alcohol-related consequences [4, 5]. Motivational theories of alcohol use posit that individuals are motivated to use alcohol when the predicted positive affective changes from alcohol use are perceived to be more desirable than not drinking [6]. Cooper’s theory of drinking motivations asserts that *motivations to drink to cope* involve motivations to reduce, avoid, or manage negative affect, while *motivations to drink to socialize* involve motivations to gain social rewards or facilitation [7]. Drinking to cope may be particularly harmful, as it has been shown to relate to higher levels of negative alcohol-related consequences, while drinking to socialize has not [8].

Identifying protective factors may help mitigate both drinking to cope and alcohol-related consequences. While some studies have focused on protective factors such as self-control [9] and triggers of alcohol use [10], one particularly important protective factor may be *trait mindfulness*, a personality characteristic referring to an individual’s tendency to be aware in the present moment without judgement [11]. Trait mindfulness is theorized to lead to other protective factors such as greater self-control by enabling individuals to nonjudgmentally observe negative thoughts and feelings without reacting to them, as well as greater awareness of triggers that lead to drinking through a higher awareness of the present moment [12]. Indeed, trait mindfulness has been linked to fewer negative alcohol-related consequences [8]. Trait mindfulness has also been shown to reduce drinking to cope with negative emotions, as it encourages a nonjudgmental acceptance of negative emotions, and therefore may reduce the need to drink to cope with overwhelming emotions [13].

More research is needed to better understand the relationship between trait mindfulness, drinking motivations, and alcohol-related consequences. Here, we propose a moderated-mediation model, where drinking motivations (drinking to cope, drinking to socialize) mediate the relationship between trait mindfulness and alcohol-related consequences, and alcohol consumption moderates the relationship between drinking motivations and alcohol-related consequences. By better understanding the protective role of trait mindfulness, researchers can better identify individuals at risk for drinking to cope and negative alcohol-related consequences. These results may also inform existing mindfulness interventions by emphasizing the relationship between mindfulness skills and drinking to cope with negative emotions in order to prevent negative alcohol-related consequences.

Below, we summarize the research on each of the four components of our moderated-mediation model: 1) trait mindfulness, 2) motivations to drink alcohol, 3) alcohol-related consequences, and 4) alcohol consumption.

### Trait Mindfulness as a Protective Factor

One factor that may prevent negative alcohol-related consequences and drinking to cope with negative emotions is trait mindfulness. While some research has focused on the impact of mindfulness-based interventions on a variety of alcohol-related outcomes [14–16], other research has demonstrated that *trait* mindfulness, an individual difference variable, is important as well [17, 18]. Trait mindfulness has been favorably related to many indices of mental and physical health, including fewer negative alcohol-related consequences, less engagement in impulsive drinking-related behaviors, and lower rates of substance use disorder [13, 19, 20].

Trait mindfulness may also protect against drinking to cope with overwhelming emotions, as individuals higher in trait mindfulness may have more nonjudgmental acceptance of negative emotions and therefore may be less likely to drink to relieve such emotions [21, 22]. For example, in a sample of 207 undergraduate problematic drinkers, drinking to cope mediated the relationship between trait mindfulness and higher levels of problematic drinking patterns [22]. In another sample of 297 undergraduates, lower levels of mindfulness predicted higher scores on the motivations to drink to cope subscale which in turn predicted more alcohol use and negative alcohol-related consequences [8].

### Motivations to Drink Alcohol

Motivations to drink to cope, compared to drinking to socialize, is not only related to trait mindfulness, but also to alcohol-related consequences. Research suggests drinking to cope is related to negative alcohol-related consequences while drinking to socialize is not [23]. For example, in a large sample of adolescents, drinking to cope was related to prevalence of alcohol-related problems while motivations of drinking to socialize were not [7]. The authors theorized that drinking to cope with negative emotions led to a deficit in healthier coping strategies and an increased reliance on alcohol to regulate overwhelming emotions. Indeed, drinking to cope has been shown to be predicted by both negative affect [24] and depressive symptoms [25] as well the expectation that drinking will alleviate negative affect [26]. Research has shown that even when controlling for consumption levels, drinking to cope is positively related to negative alcohol-related consequences [27, 28] while drinking is socialize is not [29]. Coping motives are also related to poor self-care, impaired control, and diminished self-perception [28].

Drinking to cope may not just be harmful in the short-term; this motivation for alcohol use has been related to long-term difficulties. Students who drink to cope may have trouble “maturing out” of heavy drinking patterns if their use is reinforced by a reduction of negative affect after drinking. They may forgo learning more adaptive coping strategies, leading to long-term health problems as well as substance dependency [30]. For example, in a large sample of adults, higher baseline motivations to drink to cope predicted more drinking problems up to 15 years later [31]. In contrast, drinking to socialize has been shown to be less problematic, is more commonly reported among college students [8, 32] and has been shown to be unrelated to later alcohol use/dependency [29].

### Alcohol-Related Consequences

Though research has shown that drinking to cope with negative emotions mediates the relationship between trait mindfulness and alcohol-related consequences, these studies have focused only on *negative* alcohol-related consequences. However, to gain a more nuanced understanding of how trait mindfulness and drinking motivations are related to alcohol-related consequences, researchers have begun to expand their conceptualization and measurement of alcohol-related consequences. For example, Park (2004) was the first to examine *positive* alcohol-related consequences in college students and found positive consequences to be more frequent than negative consequences, and that positive consequences reinforced their positive expectancies of alcohol use, which could in turn predict future alcohol use, indicating the importance of including positive consequences in future research [33].

Negative and positive alcohol-related consequences could also have different latent factors within or across them, which could share different relationships to trait mindfulness and drinking motivations. For example, negative alcohol-related consequences include consequences that are more normative and minor, such as waking up with a hangover, to more severe consequences, such as sustaining an injury or having trouble with the police. These negative consequences may constitute different latent factors. Evidence of likely latent structures is present in the extant literature, as one study of college students found that certain consequences typically presented as “negative” in the alcohol-related consequences literature, such as having a hangover, were viewed as neutral (27.8% of the sample) or even positive (24.9%), while consequences such as being arrested were more commonly perceived as negative (92.5%) [34]. This finding suggests less severe negative consequences could represent a separate latent factor from more severe consequences if they are viewed differently by students. Another study of first-year undergraduate students utilized latent class analysis and found that their sample could be categorized into four different classes in regards to negative alcohol-related consequences: 1) very few or no negative alcohol-related consequences; 2) academic problems, 3) injured self, and 4) severe problems [35]. Thus, while literature suggests there may be distinct subfactors of alcohol-related consequences, no study has examined the factor structure of the Positive and Negative Consequences of Alcohol Scale (PNCAS) [33] and tested whether these subfactors have differential relationships to trait mindfulness and drinking motivations.

### Alcohol Consumption

Alcohol consumption may moderate the relationship between drinking motivations and alcohol-related consequences. Drinking to cope has been related to higher levels of alcohol consumption compared to those who do not drink to cope [36] and drinking to cope has been shown to correlate with number of drinks consumed while drinking to socialize does not [8, 36]. The average number of drinks per day and frequency of drinking days were associated with greater likelihood of membership in three classes of negative alcohol-related consequences (academic problems, injured self, and severe problems) in Rinker and colleagues’ (2016) latent class analysis of undergraduates [35]. While a previous study found that alcohol consumption moderated the relationship between perceived drinking norms and alcohol-related consequences [37], no research has examined alcohol consumption as a moderator of drinking motivations and alcohol-related consequences.

### Purpose of Present Project

**Fig. 1:**
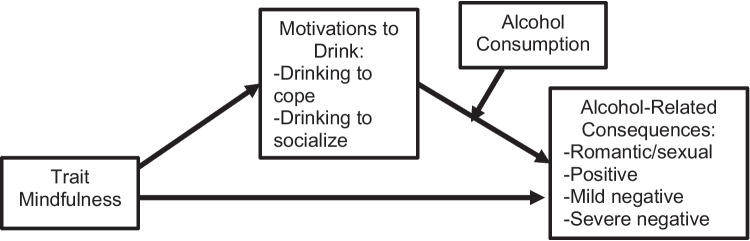
Theoretical moderated mediation model; motivations to drink mediating the relationship between trait mindfulness and alcohol-related consequences

The present study tested a moderated-mediation model to determine if drinking motivations mediate the relationship between trait mindfulness and alcohol-related consequences and whether alcohol consumption moderates the relationship between drinking motivations and alcohol-related consequences (see conceptual Fig. 1). The current study aims were:

1. Determine the factor structure of the PNCAS [33] through exploratory and confirmatory factor analysis (EFA, CFA) in a sample of undergraduates. Hypothesis: The factor structure will demonstrate more latent factors than simply positive and negative factors.
2. Using the factors of the PNCAS identified in Aim 1, we will test a moderated mediation model to determine if:
  (A) Drinking motivations (drinking to cope, drinking to socialize) mediate the relationship between trait mindfulness and the alcohol-related consequences factors. Hypothesis: Drinking to cope will mediate the relationship between mindfulness and any alcohol-related consequences that are *negative* in nature, while drinking to socialize will not as it will not relate to negative alcohol-related consequences. Hypotheses regarding *positive* alcohol-related consequences will be exploratory in nature due to a lack of previous research. Hypotheses regarding alcohol-related consequences that could be considered *both positive and negative* will also be exploratory in nature due to a lack of previous research.
  (B) Alcohol consumption moderates the relationship between drinking motives and the alcohol-related consequences factors. Hypothesis: For those who consume more alcohol, the relationship between drinking motivations and alcohol-related consequences will be stronger.

## Study 1

The purpose of the first study was to perform exploratory factor analysis on the Positive and Negative Consequences of Alcohol Scale [33] to determine the measure’s factor structure. We utilized a cross-sectional sample of undergraduates and selected only students who reported consuming alcohol in the past month.

### Materials and Methods

Students from a large public institution in the northeast participated in this study to earn class credit for introductory psychology courses. Recruitment and data collection were conducted through an anonymous university participant pool. From a larger dataset (n = 230), we examined only students who reported drinking at least one alcoholic beverage in the past 30 days (i.e., those who responded “yes” to “In the past 30 days, have you had an alcohol drink?”; n = 165). All exploratory factor analyses were conducted with Mplus [38] using maximum likelihood (ML) extraction and 1000 iterations.

### Results

Sample demographics are presented in Supplementary Materials Table 1. The sample (n = 165) was mostly White (63.6%) and female (56.9%) with a mean age of 18.33 (SD = 1.32) years. Almost 75% of the sample reported binge drinking in the past 30 days and 37% reported binge drinking in the past 7 days.

**Table 1:** Sample demographics

**Characteristic**	***N***	**Percentage**
Gender		
Female	210	70.9%
Male	62	20.9%
Transgender Female	1	0.3%
No response	23	7.8%
Race		
White	207	69.9%
Black or African	10	3.4%
American		
Asian	31	10.5%
More than one race	21	7.1%
No response	27	9.1%
Age	M = 19.04, SD = 1.25	
Alcohol Consumption		
More than 5 drinks at one time in the past 30 days	200	67.6%
More than 5 drinks at one time in the past 7 days	116	39.2%

The scree plot (eigenvalue > 1.0) indicated a four-factor fit. The fit of the four-factor model was good (Chi-Square = 485.61, df = 167, p < .001, RMSEA = .089, CFI = .91, TLI = .86, SRMR = .04). However, six items demonstrated high cross-loadings (“miss class”, “behind in school”, “forget where you were”, “forgot school problems”, “not using protection”, “performed tasks better”). The EFA was re-run with these six items removed; this revised four-factor model demonstrated good fit as well (Chi-Square = 217.05, df = 87, p < .001, RMSEA = .089, CFI = .95, TLI = 0.92, SRMR = .03). See Supplementary Materials Table 2 for final items and loadings. The four factors were: 1) romantic/sexual consequences (“more romantic”, “unplanned sex”); 2) positive (non-romantic) consequences (“felt ‘cool’”, “better expression”); 3) mild negative consequences (“had a hangover”, “argue with friends”); and 4) severe negative consequences (“damage property”, “trouble with police”, “got hurt or injured”).

**Table 2:** Scale/item descriptive statistics

**Scale**	**Total Sample**		**Women**		**Men**		***t* (women vs. men)**
	**M (SD)**	**Range**	**M (SD)**	**Range**	**M(SD)**	**Range**	
Trait mindfulness	3.16 (0.45)	1.87 – 4.53	3.18 (0.47)	1.87 – 4.53	3.10 (0.40)	2.00 – 4.20	1.12
Drinking to cope	2.02 (0.94)	1.00 – 5.00	2.03 (0.94)	1.00 – 5.00	2.00 (0.89)	1.00 – 4.20	0.03
Drinking to socialize	3.25 (1.01)	1.00 – 5.00	3.23 (1.01)	1.00 – 5.00	3.22 (1.02)	1.00 – 5.00	-0.35
Days consumed alcohol in past month	5.43 (3.37)	1.00- 20.00	5.53 (3.41)	1.00–20.00	5.28 (3.23)	1.00–16.00	-0.82
Drinks consumed on average per drinking day	5.68 (2.24)	1.00 -12.00	5.26 (1.96)	1.00–11.00	6.78 (2.41)	1.00–12.00	4.91
Drinks per month	33.89 (27.56)	1.00 – 150.00	32.37 (26.58)	1.00 – 150.00	38.55 (28.92)	1.00 – 128.00	1.32
Romantic/sexual consequences	1.70 (0.89)	1.00 – 5.00	1.65 (0.86)	1.00 – 5.00	1.58 (0.86)	1.00 – 4.67	0.42
Positive consequences	1.90 (0.75)	1.00 – 4.38	1.88 (0.74)	1.00 – 4.38	1.88 (0.77)	1.00 – 3.88	-0.42
Mild negative consequences	1.69 (0.70)	1.00 – 4.33	1.67 (0.68)	1.00 – 3.67	1.68 (0.72)	1.00 – 4.33	-0.62
Severe negative consequences	1.13 (0.33)	1.00 – 3.75	1.11 (0.32)	1.00 – 3.75	1.16 (0.36)	1.00 – 3.25	-1.08

*p < .001

## Study 2

The purpose of Study 2 was to: 1) confirm the factor structure of the Positive and Negative Consequences of Alcohol Scale in a new sample of undergraduates, 2) test whether drinking motivations mediated the relationship between trait mindfulness and alcohol consequences and whether the amount of alcohol consumed moderated the relationship between drinking motives and alcohol-related consequences (see Fig. 1 for conceptual model).

### Methods and Materials

#### Procedure/Sample

Students from a large public institution in the northeast participated in this study to earn class credit for introductory psychology courses. Recruitment and data collection were conducted through an anonymous university participant pool. From a larger dataset (n = 440), we examined only students who reported drinking at least one alcoholic beverage in the past 30 days (i.e., those who responded “yes” to “In the past 30 days, have you had an alcohol drink?”; n = 296).

#### Measures

Motivations to drink were measured with the drinking to cope and drinking to socialize subscales of the Motivations for Alcohol Use Scale [7]. The instructions given were, “Thinking of all the times you drink, how often would you say that you drink for each of the following reasons?” Items such as, “To forget about your problems” (drinking to cope) and “To be sociable” (drinking to socialize) were rated on a 1 (*Never/almost never*) to 5 (*Almost always/always*) scale. The reliabilities in the current sample were very good (drinking to cope α = .91; drinking to socialize α = .89).

Trait mindfulness was measured with the Five Facet Mindfulness Questionnaire (FFMQ) [39]. The sum score of the FFMQ was used in the current analyses. Participants rated items such as, “I think some of my emotions are bad or inappropriate and I shouldn’t feel them” on a 1 (*Never or very rarely true*) to 5 (*Very often or always true*) scale. The internal consistency reliability in the current sample was acceptable (α = .69).

Alcohol-related consequences were measured with the 23-item Positive and Negative Consequences of Alcohol Scale (PNCAS) [33], a compilation of items from the Negative Consequences Scale [40] and adapted alcohol outcome expectancy items [41]. Participants rated the consequences experienced in the past two months as a result of alcohol use on a 1 (*Never*) to 5 (*Very frequently*) scale. Items spanned negative consequences (e.g., “having a hangover” and “trouble with police”) as well as positive consequences (e.g., “felt relaxed” and “fit in with people”).

Drinks consumed per month was estimated by asking participants how many days they drank in the past month (1–31) and how many drinks on average they consumed each time they drank and multiplying these numbers together.

#### Analyses

Frequency analysis was performed for gender, race, and alcohol consumption, and the mean and standard deviation were calculated for age. Descriptive statistics (means, standard deviations, ranges) were performed for trait mindfulness, motivations to drink (to cope, to socialize), drinks per month, and alcohol-related consequences. Descriptive statistics are reported for the entire sample as well as women and men separately. T-tests were used to compare group means between women and men.

EFA was performed on a separate sample of undergraduates (n = 165) who were recruited in the same manner as detailed above. See Supplemental Materials for participant demographics, a full description of the methods, and factor loadings. Four factors were found: 1) romantic/sexual consequences (“more romantic”, “unplanned sex”); 2) positive (non-romantic) consequences (“felt ‘cool”, “better expression”); 3) mild negative consequences (“had a hangover”, “argue with friends”); and 4) severe negative consequences (“damage property”, “trouble with police”, “got hurt or injured”). Items that did not load significantly onto a factor or that demonstrated high cross-loadings were not included in the current study. All CFAs were conducted with Mplus using the ML estimator [38] and 1000 iterations.

Eight regression models were used to determine whether drinking motivations (drinking to cope, drinking to socialize) mediated the relationship between trait mindfulness and the four alcohol-related consequences factors, and whether number of drinks consumed in the past month moderated the relationship between drinking motivations and alcohol-related consequences. Regression models were run with the PROCESS module in SPSS [42]. Variables were mean-centered and indirect effects were tested using a bootstrap estimation approach with 5,000 samples and bias-corrected estimates.

#### Power Analysis

To ensure that our sample was sufficiently powered, we conducted a sensitivity power analysis for our moderated-mediation models using WebPower [43]. With a sample size of 296, the current study was deemed to be sufficiently powered to detect small effect sizes (F2 = 0.15).

### Results

#### Sample Demographics

See Table 1 for sample demographics. The sample was primarily White (69.9%) and female (70.9%) with a mean age of 19.04 (SD = 1.25) years. Approximately 68% of the sample reported binge drinking in the past 30 days and 39.2% reported binge drinking in the past 7 days.

#### Descriptive Statistics

Descriptive statistics for drinking motivations, alcohol-related consequences, trait mindfulness, and drinks per month are presented in Table 2. Students reported higher motivations to drink to socialize than to drink to cope. Mean scale scores on the alcohol-related consequences were highest for positive consequences. Participants reported drinking a mean of 5.43 (SD = 3.37) days in the past month and a mean of 5.68 (SD = 2.24) number of drinks per drinking session and a mean of 33.89 (SD = 27.56) estimated drinks in the past month. Means on all of these variables were computed for women and men separately; independent t-tests were used to compare group means (see Table 2). Women reported significantly fewer drinks per drinking day (M = 5.26) than men (M = 6.78; *t* = 4.91, p < .01); otherwise, women’s and men’s means did not differ across the variables of interest.

#### Correlations

Correlations among trait mindfulness, drinking to cope and to socialize, the four alcohol-related consequences factors, and drinks per month are presented in Table 3. Drinks per month was positively related to all four types of alcohol-related consequences (*r*’s from .32 – .53; p’s < .01) as well as both drinking to cope (*r* = .43, p < .01) and drinking to socialize (*r* = .39, p < .01). Higher levels of trait mindfulness were related to lower levels of drinking to cope (*r* = -.28, p < .01) but were not related to drinking to socialize. Drinking to cope was positively correlated with all four of the alcohol related-consequences scales (*r*’s from .30  – .57; p’s < .01) as was drinking to socialize (*r*’s from .17 – .55; p’s < .01).

#### Factor Analyses

**Table 3:** Correlations between variables in moderated-mediation model

	1	2	3	4	5	6	7
1. Mindfulness							
2. Drinking to cope	-.28**						
3. Drinking to socialize	-.07	.53**					
4. Drinks per month	-.15*	.43**	.39**				
5. Romantic/sexual cons	-.11	.44**	.39**	.49**			
6. Positive cons	-.07	.57**	.55**	.44**	.57**		
7. Mild negative cons	-.18**	.52**	.45**	.53**	.58**	.53**	
8. Severe cons	-.07	.30**	.17**	.32**	.37**	.37**	.43**

*p < .05; **p < .01

CFA was used with the current sample to confirm the factor structure in a different sample of students. The overall fit of the four-factor model was acceptable (Chi-Square = 400.42, df = 129, p < .001, RMSEA = .083, 90% CI = .074 – .092, CFI = .88, TLI = .85, SRMR = .07). All factor loadings were greater than .40.

#### Drinking to Cope Moderated Mediation Models

Regression analyses indicated that drinking to cope significantly mediated the relationship between trait mindfulness and all four alcohol-related consequences. See Supplemental Table 3 and Fig. 1  for results of the moderated mediation models.

##### Romantic and Sexual Consequences

Trait mindfulness significantly predicted drinking to cope (B = -3.01, p < .001). Drinking to cope significantly predicted romantic/sexual consequences (B = 0.05, p < .001), and trait mindfulness did not significantly predict romantic/sexual consequences, indicating complete mediation. The indirect effect was significant (B = -0.24, SE = 0.06, 90% CI = -0.37 – -0.13) and the percentage of the mediation was 88%. Drinks per month was not a significant moderator of the relationship between drinking to cope and romantic/sexual consequences. However, number of drinks per month did significantly predict romantic/sexual consequences (B = 0.01, p < .001).

##### Positive Consequences

Trait mindfulness significantly predicted drinking to cope (B = -3.01, p < .001). Drinking to cope significantly predicted positive consequences (B = 0.08, p < .001), and trait mindfulness predicted positive consequences (B = 0.17, p = .04). The indirect effect was significant (B = -0.27, SE = 0.06, 90% CI = -0.39 – 0.16) and the percentage of the mediation was 64%. Drinks per month was not a significant moderator of the relationship between drinking to cope and positive consequences. However, number of drinks per month did significantly predict positive consequences (B = 0.01, p < .001).

##### Mild Negative Consequences

Trait mindfulness significantly predicted drinking to cope (B = -3.01, p < .001). Drinking to cope significantly predicted mild negative consequences (B = 0.05, p < .001), and trait mindfulness did not significantly predict mild negative consequences, indicating complete mediation. The indirect effect was significant (B = -0.22, SE = 0.05, 90% CI = -0.31 – -0.13) and the percentage of the mediation was 78%. Drinks per month was a significant moderator of the relationship between drinking to cope and mild negative consequences (B = 0.0002, p = .01), such that those who consumed more drinks per month demonstrated a stronger relationship between drinking to cope and mild negative consequences. Number of drinks per month also significantly predicted mild negative consequences (B = 0.01, p < .001).

##### Severe Negative Consequences

Trait mindfulness significantly predicted drinking to cope (B = -3.01, p < .001). Drinking to cope significantly predicted severe negative consequences (B = 0.01, p < .001), and trait mindfulness did not significantly predict severe negative consequences, indicating complete mediation. The indirect effect was significant (-0.06, SE = 0.02, 90% CI = -0.10 – -0.02) and the percentage of the mediation was 83%. Number of drinks per month was not a significant moderator of the relationship between drinking to cope and severe negative consequences. However, number of drinks per month significantly predicted severe negative consequences (B = 0.003, p < .001).

#### Drinking to Socialize Moderated Mediation Models

Regression analyses indicated that drinking to socialize did not significantly mediate the relationship between trait mindfulness and all four alcohol-related consequences, as the A path of the mediation model (trait mindfulness to drinking to socialize) was not significant (p = .08). See Supplemental Table 4 and Fig. 2 for results of the moderated mediation models.

##### Romantic and Sexual Consequences

Trait mindfulness significantly predicted drinking to cope (B = -3.01, p < .001). Drinking to cope significantly predicted romantic/sexual consequences (B = 0.05, p < .001), and trait mindfulness did not significantly predict romantic/sexual consequences, indicating complete mediation. The indirect effect was significant (B = -0.24, SE = 0.06, 90% CI = -0.37 – -0.13) and the percentage of the mediation was 88%. Drinks per month was not a significant moderator of the relationship between drinking to cope and romantic/sexual consequences. However, number of drinks per month did significantly predict romantic/sexual consequences (B = 0.01, p < .001).

##### Positive Consequences

Trait mindfulness significantly predicted drinking to cope (B = -3.01, p < .001). Drinking to cope significantly predicted positive consequences (B = 0.08, p < .001), and trait mindfulness predicted positive consequences (B = 0.17, p = .04). The indirect effect was significant (B = -0.27, SE = 0.06, 90% CI = -0.39 – 0.16) and the percentage of the mediation was 64%. Drinks per month was not a significant moderator of the relationship between drinking to cope and positive consequences. However, number of drinks per month did significantly predict positive consequences (B = 0.01, p < .001). 

##### Mild Negative Consequences

Trait mindfulness did not predict drinking to socialize (B = -1.59, p = .08). Drinking to socialize significantly predicted mild negative consequences (B = 0.04, p < .001), and trait mindfulness did significantly predict mild negative consequences. The indirect effect was not significant (B = -0.01, SE = 0.04, 90% CI = -0.08 – 0.08). Number of drinks per month was not a significant moderator of the relationship between drinking to socialize and mild negative consequences. However, number of drinks per month did significantly predict mild negative consequences (B = 0.01, p < .001).

##### Severe Negative Consequences

Trait mindfulness did not predict drinking to socialize (B = -1.59, p = .08). Drinking to socialize did not significantly predict severe negative consequences, and trait mindfulness did not significantly predict severe negative consequences. The indirect effect was not significant (B = -0.001, SE = 0.01, 90% CI = -0.01 – 0.0). Number of drinks per month was not a significant moderator of the relationship between drinking to socialize and severe negative consequences. However, number of drinks per month did significantly predict severe negative consequences (B = 0.001, p < .001).

## Discussion

These results provide several contributions to research examining relationships between trait mindfulness, motivations to drink, and alcohol-related consequences. First, factor analysis was used to determine the factor structure of the Positive and Negative Alcohol-Related Consequences Scale [33] in order to measure alcohol-related consequences in a more nuanced manner. Results supported Hypothesis 1; exploratory and confirmatory factor analyses provided support for four factors (romantic/sexual consequences, positive consequences, mild negative consequences, and severe negative consequences). Separate factors existed within the negative consequences realm, and this distinction allowed us to determine that drinking to cope, but not drinking to socialize, was related to severe negative consequences. Continuing to examine alcohol-related consequences in a more nuanced way will allow researchers and clinicians to better distinguish between individuals at risk of severe versus mild negative alcohol-related consequences. Additionally, the romantic/sexual consequences factor contained both positive and negative consequence items. This suggests that some college students may find certain outcomes, such as unplanned sex, to be either positive or negative.

This study also demonstrated that drinking to cope mediated the relationship between trait mindfulness and the four alcohol-related consequences, providing support for Hypothesis 2a. This set of findings builds upon previous research showing that drinking to cope mediated the relationship between trait mindfulness and only alcohol-related *problems* [8] as the current study demonstrated that drinking to cope predicted a range of consequences, including *positive* consequences. Thus, it may be the case that individuals who tend to drink to cope both lack more adaptive, healthy coping strategies that could lead to negative consequences *and* experience reinforcement of this coping style from subsequent positive alcohol-related consequences.

As expected in Hypothesis 2a, drinking to socialize was not a significant mediator in the moderated mediation models. Trait mindfulness did not significantly predict drinking to socialize in all four models, and drinking to socialize was only related to three of the alcohol-related consequences and did not predict severe alcohol-related consequences. This finding suggests that drinking to cope may be more problematic than drinking to socialize in that it not only tends to have stronger relationships to negative alcohol-related consequences [27, 28], but is particularly relevant in terms of *severe* negative consequences, such as getting in trouble with the police or getting injured. This relationship between drinking to cope and severe consequences may be due to the fact that individuals who tend to drink to cope with overwhelming emotions tend to lack adaptive, healthy coping strategies in general and tend to have impaired self-control and poorer self-care [28], putting them at greater risk of more severe consequences such as getting hurt or injured. However, further research is needed to support this explanation, as measures of coping, self-control and self-care were not included in the current study.

Contrary to Hypothesis 2b, we did not find evidence that the number of drinks consumed per month moderated the relationship between either drinking to cope and drinking to socialize and the alcohol-related consequences factors except for that between drinking to cope and mild negative consequences, such that high levels of alcohol use strengthened the relationship between drinking to cope and mild negative consequences. As drinking amount was only a significant moderator in one of eight models, it appears that this variable is not of great importance when considering the relationship between drinking motivations and consequences. When predicting severe negative consequences, it may matter more whether an individual is drinking to cope, and therefore may lack more adaptive coping strategies, or is drinking to socialize, and therefore may possess higher levels of adaptive coping strategies, rather than considering how much alcohol the individual consumes.

### Clinical Implications

These results found that lower levels of trait mindfulness predicted more drinking to cope but did not predict drinking to socialize. This indicates that interventions for this population should aim to teach college students mindfulness skills to order to view negative emotions in a nonjudgmental, accepting way in order to reduce the need to use alcohol to cope, which may ultimately reduce severe negative consequences. Interestingly, this study also found that trait mindfulness was related to positive alcohol-related consequences. Individuals higher in trait mindfulness may be more nonjudgmentally aware of both the positive and negative results of alcohol use and be able to use alcohol in a more controlled manner in order to obtain more positive results. Future mindfulness interventions should therefore also consider emphasizing more adaptive ways to achieve positive results (e.g., relaxation techniques to reduce anxiety) rather than using alcohol.

### Limitations

This study is limited in several ways. First, these data were collected at the same time in studies cross-sectional in design; thus, neither causality nor even temporality can be inferred. For example, it is unclear whether drinking to cope actually causes negative alcohol-related consequences or whether both drinking to cope and negative consequences may be predicted by a general lack of adaptive coping strategies. Second, the measurement of motivations to drink alcohol asked students to identify how often they drink to cope or drink to socialize while considering all times they have drank, while alcohol-related consequences of alcohol were measured over the past two months. Thus, we were unable to capture whether a specific incident of drinking was motivated by a need to cope and led to specific negative alcohol-related consequences. Third, this sample consisted primarily of White women who are college freshmen; it is unclear how these relationships hold for more racially diverse groups and older college students. Fourth, we conceptualized drinking to cope and drinking to socialize as distinct factors in this study. However, research has demonstrated that individuals may drink to cope with social anxiety [44], indicating that there may be significant overlap in these factors which was not accounted for in this study.

### Future Directions

Based on these results, future research should aim to examine alcohol-related consequences in a more nuanced way rather than simply negative and positive consequences, as this study found that mild and severe negative consequences were two separate factors and severe negative consequences had distinct relationships with drinking to cope and drinking to socialize. More research is also needed to determine if certain consequences, such as unplanned sex, are viewed differently by gender. Future research can further clarify causality by examining these variables longitudinally by using a daily diary approach. Additionally, a more diverse sample in terms of race and gender would allow researchers to make better inferences about how these relationships hold across various groups; for example, some research has indicated that racial discrimination may cause racial and ethnic minorities to consume more alcohol and experience more negative alcohol-related consequences [45]. Researchers should also aim to collect samples with participants from all years of college (i.e., freshman through senior), as research has shown that older college students may be less likely to experience alcohol-related consequences compared to first-year college students [46]. Future research should also determine how different facets of mindfulness predict drinking motivations and alcohol-related consequences. Finally, measuring coping behavior will help to explain the nature of several of these relationships.

Overall, this study provides evidence for researchers to measure alcohol-related consequences in a nuanced manner, to consider the importance of what is motivating a student to engage in alcohol use, and to consider that mindfulness interventions may be particularly useful for students who tend to drink to cope with overwhelming emotions. By continuing to explore the associations among these factors, interventions can be modified for specific purposes and populations in order to decrease the rate of negative alcohol-related consequences and perhaps even long-term consequences such as alcohol use disorders.

## Supplementary Information

Below is the link to the electronic supplementary material.

Supplementary file1 (DOCX 127 KB)

## Data Availability

The data that support the findings of this study are available from the corresponding author, LC, upon reasonable request.
