# Supplementary material for: Alcohol-related Consequences: Factor Structure and Associations With Trait Mindfulness and Drinking Motivations
Source: Int J Behav Med. 2024 Jun 4;32(4):550–60. doi: 10.1007/s12529-024-10300-7 (PMC12328501; doi:10.1007/s12529-024-10300-7)
Supplement: Supplementary file 1 — Supplementary file1 (DOCX 127 KB) [file 12529_2024_10300_MOESM1_ESM.docx]

**Appendix**

Supplemental Table 1

*Participant demographics*

| **Characteristic** | ***N*** | **Percentage** |
| --- | --- | --- |
| Gender |  |  |
| Female | 94 | 56.9% |
| Male | 61 | 37.0% |
| Transgender Female | 0 | 0% |
| No response | 10 | 6.1% |
| Race |  |  |
| White | 105 | 63.6% |
| Black or African  American | 10 | 6.1% |
| Asian | 24 | 14.5% |
| More than one race | 12 | 7.2% |
| No response | 14 | 8.5% |
| Age | M = 18.33, SD = 1.32 |  |
| Alcohol Consumption |  |  |
| More than 5 drinks at  one time in past 30  days | 123 | 74.5% |
| More than 5 drinks at  one time in past 7 days | 61 | 37.0% |

Supplemental Table 2

*Positive and negative alcohol-related consequences scale EFA loadings*

| **Items** | **Romantic/sexual consequences** | **Positive consequences** | **Mild negative consequences** | **Severe negative consequences** |
| --- | --- | --- | --- | --- |
| Unplanned sex | 0.46* | 0.01 | 0.12 | 0.39* |
| More romantic | 0.84* | 0.01 | 0.05 | -0.01 |
| More relaxed about sex | 0.80* | 0.07 | 0.05 | 0.05 |
| Felt “cool” | -0.05 | 0.54* | 0.18* | 0.17* |
| More creativity | 0.16 | 0.51* | -0.08 | 0.28* |
| Felt relaxed | 0.18 | 0.69* | -0.01 | -0.19* |
| Better expression | 0.14 | 0.71* | 0.03 | -0.02 |
| Felt better about self | -0.15 | 0.89* | 0.02 | -0.01 |
| Fit in with people | 0.00 | 0.82* | 0.03 | 0.05 |
| Added enjoyment to a meal | 0.12 | 0.48* | -0.12 | 0.01 |
| Have a hangover | 0.07 | -0.02 | 0.63* | -0.07 |
| Regret something | 0.09 | 0.06 | 0.72* | 0.05 |
| Argue with friends | 0.01 | 0.03 | 0.52* | 0.45* |
| Damage property | 0.27* | 0.03 | -0.06 | 0.68* |
| Trouble with police | -0.02 | -0.03 | 0.05 | 0.75* |
| Got hurt or injured | 0.02 | 0.21* | 0.14 | 0.55* |
| Overdose | -0.02 | -0.07 | -0.04 | 0.78* |

Note: * p < .05

Supplemental Table 3

*Drinking to cope moderated-mediation models*

| **Sexual/Romantic Consequences** | | | | | | |
| --- | --- | --- | --- | --- | --- | --- |
| **Predicting Drinking to Cope** | | | | | | |
| **Predictors** | **B** | **SE** | **Lower CI** | **Upper CI** | ***p*** | **R^2^** |
| Trait mindfulness | -3.01 | 0.59 | -4.17 | -1.85 | < 0.001 | 0.090 |
| **Predicting Sexual/Romantic Consequences** | | | | | | |
| Trait mindfulness | 0.05 | 0.10 | -0.14 | 0.25 | 0.64 | 0.310 |
| Drinking to cope | 0.05 | 0.01 | 0.03 | 0.06 | < 0.001 |  |
| Drinks per month | 0.01 | 0.01 | 0.01 | 0.02 | < 0.001 |  |
| Drinking to cope * drinks per month | < 0.001 | < 0.001 | <- 0.001 | < 0.001 | 0.62 |  |
| **Total, Direct, and Indirect Effects of Mediation Model** | | | | | | |
| Total Effect | -0.21 | 0.12 | 0.07 | -0.44 | -0.11 |  |
| Direct Effect | 0.03 | 0.11 | -0.18 | 0.25 | 0.77 |  |
| Indirect Effect | -0.24 | 0.06 | -0.37 | -0.13 |  |  |
| **Positive Consequences** | | | | | | |
| **Predicting Drinking to Cope** | | | | | | |
| **Predictors** | **B** | **SE** | **Lower CI** | **Upper CI** | ***p*** | **R^2^** |
| Trait mindfulness | -3.01 | 0.59 | -4.17 | -1.85 | < 0.001 | 0.090 |
| **Predicting Positive Consequences** | | | | | | |
| Trait mindfulness | 0.17 | 0.08 | 0.01 | 0.32 | 0.04 | 0.379 |
| Drinking to cope | 0.08 | 0.01 | 0.06 | 0.10 | < 0.001 |  |
| Drinks per month | 0.01 | 0.01 | 0.001 | 0.003 | 0.01 |  |
| Drinking to cope * drinks per month | < 0.001 | < 0.001 | -0.001 | < 0.001 | 0.28 |  |
| **Total, Direct, and Indirect Effects of Mediation Model** | | | | | | |
| Total Effect | -0.12 | 0.10 | -0.31 | 0.07 | 0.22 |  |
| Direct Effect | 0.15 | 0.08 | -0.01 | 0.31 | 0.07 |  |
| Indirect Effect | -0.27 | 0.06 | -0.39 | -0.16 |  |  |
| **Mild Negative Consequences** | | | | | | |
| **Predictors** | **B** | **SE** | **Lower CI** | **Upper CI** | ***p*** | **R^2^** |
| Trait mindfulness | -3.01 | 0.59 | -4.17 | -1.85 | < 0.001 | 0.090 |
| **Predicting Mild Negative Consequences** | | | | | | |
| Trait mindfulness | -0.05 | 0.07 | -0.19 | 0.08 | 0.44 | 0.218 |
| Drinking to cope | 0.05 | 0.01 | 0.04 | 0.07 | < 0.001 |  |
| Drinks per month | 0.01 | 0.001 | 0.01 | 0.012 | < 0.001 |  |
| Drinking to cope * drinks per month | < 0.001 | < 0.001 | <0.001 | <0.001 | 0.01 |  |
| **Total, Direct, and Indirect Effects of Mediation Model** | | | | | | |
| Total Effect | -0.27 | 0.09 | -0.44 | -0.10 | 0.002 |  |
| Direct Effect | -0.06 | 0.08 | -0.21 | 0.10 | 0.48 |  |
| Indirect Effect | -0.22 | 0.05 | -0.31 | -0.13 |  |  |
| **Severe Negative Consequences** | | | | | | |
| **Predictors** | **B** | **SE** | **Lower CI** | **Upper CI** | ***p*** | **R^2^** |
| Trait mindfulness | -3.01 | 0.59 | -4.17 | -1.85 | < 0.001 | 0.090 |
| **Predicting Severe Negative Consequences** | | | | | | |
| Trait mindfulness | 0.01 | 0.04 | -0.07 | 0.09 | 0.73 | .218 |
| Drinking to cope | 0.01 | 0.004 | 0.01 | 0.02 | 0.002 |  |
| Drinks per month | 0.003 | 0.001 | 0.002 | 0.004 | < 0.001 |  |
| Drinking to cope * drinks per month | <0.001 | <0.001 | <0.001 | <0.001 | 0.18 |  |
| **Total, Direct, and Indirect Effects of Mediation Model** | | | | | | |
| Total Effect | -0.05 | 0.04 | -0.13 | 0.03 | 0.24 |  |
| Direct Effect | 0.01 | 0.04 | -0.07 | 0.09 | 0.76 |  |
| Indirect Effect | -0.06 | 0.02 | -0.10 | -0.02 |  |  |

Supplemental Table 4

*Drinking to socialize moderated-mediation models*

| **Romantic Consequences** | | | | | | |
| --- | --- | --- | --- | --- | --- | --- |
| **Predicting Drinking to Cope** | | | | | | |
| **Predictors** | **B** | **SE** | **Lower CI** | **Upper CI** | ***p*** | **R^2^** |
| Trait mindfulness | -1.59 | 0.89 | -3.35 | 0.17 | 0.08 | 0.02 |
| **Predicting Sexual/Romantic Consequences** | | | | | | |
| Trait mindfulness | -0.20 | 0.13 | -0.47 | 0.06 | 0.12 | 0.25 |
| Drinking to socialize | 0.01 | 0.004 | 0.005 | 0.02 | 0.002 |  |
| Drinks per month | 0.003 | 0.001 | 0.001 | 0.002 | < 0.001 |  |
| Drinking to socialize * drinks per month | < 0.001 | < 0.001 | <- 0.001 | < 0.001 | 0.18 |  |
| **Total, Direct, and Indirect Effects of Mediation Model** | | | | | | |
| Total Effect | -0.21 | 0.11 | -0.42 | -0.01 | 0.05 |  |
| Direct Effect | -0.20 | 0.10 | -0.40 | -0.01 | 0.05 |  |
| Indirect Effect | -0.01 | 0.04 | -0.09 | 0.01 |  |  |
| **Positive Consequences** | | | | | | |
| **Predicting Drinking to Cope** | | | | | | |
| **Predictors** | **B** | **SE** | **Lower CI** | **Upper CI** | ***p*** | **R^2^** |
| Trait mindfulness | -1.59 | 0.89 | -3.35 | 0.17 | 0.08 | 0.02 |
| **Predicting Positive Consequences** | | | | | | |
| Trait mindfulness | -0.10 | 0.11 | -0.33 | 0.12 | 0.36 | 0.35 |
| Drinking to socialize | 0.06 | 0.01 | 0.05 | 0.08 | < 0.001 |  |
| Drinks per month | 0.01 | 0.001 | 0.004 | 0.01 | < 0.001 |  |
| Drinking to socialize * drinks per month | < 0.001 | < 0.001 | -0.001 | < 0.001 | 0.86 |  |
| **Total, Direct, and Indirect Effects of Mediation Model** | | | | | | |
| Total Effect | -0.23 | 0.13 | -0.49 | 0.03 | 0.07 |  |
| Direct Effect | -0.10 | 0.11 | -0.33 | 0.12 | 0.36 |  |
| Indirect Effect | -0.13 | 0.08 | -0.29 | -0.01 |  |  |
| **Mild Negative Consequences** | | | | | | |
| **Predictors** | **B** | **SE** | **Lower CI** | **Upper CI** | ***p*** | **R^2^** |
| Trait mindfulness | -1.59 | 0.89 | -3.35 | 0.17 | 0.08 | 0.02 |
| **Predicting Mild Negative Consequences** | | | | | | |
| Trait mindfulness | 0.004 | 0.11 | -0.21 | 0.23 | 0.97 | 0.25 |
| Drinking to socialize | 0.04 | 0.01 | 0.02 | 0.05 | < 0.001 |  |
| Drinks per month | 0.01 | 0.001 | 0.01 | 0.012 | < 0.001 |  |
| Drinking to socialize * drinks per month | < 0.001 | < 0.001 | <0.001 | <0.001 | 0.78 |  |
| **Total, Direct, and Indirect Effects of Mediation Model** | | | | | | |
| Total Effect | -0.27 | 0.09 | -0.44 | -0.10 | 0.002 |  |
| Direct Effect | -0.27 | 0.08 | -0.42 | -0.11 | 0.001 |  |
| Indirect Effect | -0.01 | 0.04 | -0.08 | 0.08 |  |  |
| **Severe Negative Consequences** | | | | | | |
| **Predictors** | **B** | **SE** | **Lower CI** | **Upper CI** | ***p*** | **R^2^** |
| Trait mindfulness | -1.59 | 0.89 | -3.35 | 0.17 | 0.08 | 0.02 |
| **Predicting Severe Negative Consequences** | | | | | | |
| Trait mindfulness | -0.07 | 0.07 | -0.21 | 0.07 | 0.33 | .12 |
| Drinking to socialize | 0.01 | 0.004 | -0.01 | 0.01 | 0.77 |  |
| Drinks per month | 0.003 | 0.001 | 0.002 | 0.004 | < 0.001 |  |
| Drinking to socialize * drinks per month | <0.001 | <0.001 | <0.001 | <0.001 | 0.18 |  |
| **Total, Direct, and Indirect Effects of Mediation Model** | | | | | | |
| Total Effect | -0.05 | 0.04 | -0.13 | 0.03 | 0.23 |  |
| Direct Effect | -0.05 | 0.04 | -0.13 | 0.03 | 0.23 |  |
| Indirect Effect | -0.001 | 0.01 | -0.02 | 0.01 |  |  |

Supplemental Figure 1

*Drinking to cope moderated meditation models*

Alcohol Consumption

Drinking to Cope

.0001 (ns)

-3.01 ***

Romantic /sexual consequences

0.05***

Trait Mindfulness

.05 (ns)

R^2^ = .31, p < .001

Alcohol Consumption

Drinking to Cope

.0001 (ns)

-3.01 ***

0.08***

Trait Mindfulness

Positive consequences

.17 *

R^2^ = .38, p < .001

Alcohol Consumption

Drinking to Cope

-3.01***

.0001*

0.05***

Mild

negative consequences

Trait Mindfulness

-.05 (ns)

R^2^ = .22, p < .001

Alcohol Consumption

Drinking to Cope

.0001 (ns)

-3.01 ***

0.01*

Severe negative consequences

Trait Mindfulness

.01 (ns)

R^2^ = .22, p < .001

Supplemental Figure 2

*Drinking to socialize moderated mediation models*

Alcohol Consumption

Drinking to Socialize

-1.59, p = .08

.0001 (ns)

0.01***

Romantic /sexual consequences

Trait Mindfulness

-.20 (ns)

R^2^ = .25, p < .001

Alcohol Consumption

Drinking to Socialize

-1.59, p = .08

.0001 (ns)

0.06***

Positive consequences

Trait Mindfulness

-10. (ns)

R^2^ = .35, p < .001

Alcohol Consumption

Drinking to Socialize

-1.59, p = .08

.0001 (ns)

0.04***

Mild

negative consequences

Trait Mindfulness

0.004 (ns)

R^2^ = .25, p < .001

Alcohol Consumption

Drinking to Socialize

-1.59, p = .08

.0001 (ns)

0.001 (ns)

Severe

negative consequences

Trait Mindfulness

-.02 (ns)

R^2^ = .12, ns
